# Pathways as "signatures in landscape": towards an ethnography of mobility among the *Mbya-Guaraní *(Northeastern Argentina)

**DOI:** 10.1186/1746-4269-3-2

**Published:** 2007-01-05

**Authors:** Marta Crivos, María Rosa Martínez, María Lelia Pochettino, Carolina Remorini, Anahí Sy, Laura Teves

**Affiliations:** 1 Consejo Nacional de Investigaciones Científicas y Técnicas (CONICET); 2 Facultad de Ciencias Naturales y Museo, Universidad Nacional de La Plata, Paseo del Bosque s/n – CP 1900 – La Plata, Argentina

## Abstract

Processes of spatial mobility among the *Mbya *are of interest in anthropological and ethnobiological studies, as these processes are related to transformations in the landscape and the environment. Despite this, ethnographic literature usually focuses itself on the mobility of *Guaraní *communities from the perspective of population dynamics on a regional scale.

Our research among two *Mbya-Guaraní *communities in the Argentinean province of Misiones has enabled us to recognize patterns of mobility on a micro-scale. Certainly, the mobility of adult members of these communities as they perform hunting and gathering activities delimit spaces of individual use. We consider the different pathways as "signatures in landscape", resulting from processes of spatial mobility inherent to those activities

Taking into account the gathering and circulation of medicinal plants for treatment of gastrointestinal illnesses, we have been able to identify different pathways inherent in their search, towards the *monte *or other spaces away from de settlement. The design and construction of the pathways is determined by the specific personal knowledge of individuals who search for these valuable resources.

Using both strategies of direct observation – as members of the community manipulate different resources during these search and gathering trips – and interviews, we have been able to gather and interpret significant information on the strategies used by the *Mbya *to domesticate the *monte *areas.

As a consequence of our approach we suggest that the landscape design resulting from these trips should not be considered a consensual or collective strategy of the whole community; it is rather the result of the daily strategies of individuals, which involves the selection of resources mainly based on each individual's knowledge and interests.

## 1. Background

### 1.1. The scenery of the Mbya

The *Mbya *inhabit the *Paranaense *Rainforest – among the environmental systems of South America, one of the richest in its biodiversity. The *Mbya *– together with the *Kayova *and the *Ñandeva *– is one of the ethnic groups of the *Tupí-Guaraní *linguistic family which currently inhabit this ecosystem.

From a phytogeographical perspective, the *Paranaense *province is part of the Amazon dominium – a vast area of tropical and subtropical rainforests, forests and veldts. This area takes up the Amazon basin, most of the basin of the *Paraná *River, and the Eastern slopes of the Andes mountain range in the tropical area. The *Paranaense *province takes up the whole of the Misiones province and the Northeast of the province of Corrientes in Argentina, and it continues through Brazil and the East of Paraguay.

The area of study is located to the mid-west of the Misiones province, Argentina, and is part of the Mixed Forest District, *Paranaense *Phytogeographical Province – an area of transition between the Brazilian "*planalto*" and the District of the "*Campos*." The rainforest runs all along the margins of the Paraná and Uruguay Rivers to the South, forming narrow corridors. The local climatic pattern is hot and damp, with an annual rainfall ranging between 1800 and 2000 mm (though without any distinct rainy season), and with annual average temperatures of around 20°C with a high of around 40°C [1].

The area is comprised of various ecotones, indicative of different edaphic conditions and also different ways in which the land has been used. The semi-deciduous tropical rainforest that is characteristic of the region presents a crown canopy 20–30 meters high, and has several plant species such as *urunday *(*Astronium balansae*), *ambay *(*Cecropia adenopus*), laurel trees (*Ocotea sp*., *Nectandra sp*.), *lapacho *trees (*Tabebuia spp*.), *pindó *(*Syagrus romanzzofianum*), among other species. Canes, or *takua *(*Guadua trinii*, *Chusquea spp*., *Merostachys clausenii*), are to be found in the shrubby stratum as the predominant form of vegetation. The rainforest in this area varies in make-up according to its location – along high or low gradients, according to its proximity to streams, and depending on soil composition (most of which is laterite). Due to the transitional characteristics of the area, certain differences with the so-called *Paranaense *Rainforest can be observed, notably the absence of *pino paraná *(*Araucaria araucana*) and *palmito *(*Euterpe edulis*) [2]. This environment has been modified by human intervention to different extents, which has brought about the existence of secondary tropical rainforests (in areas where the forest has been able to see a period of re-growth), *capueras *(areas which have been cleared, and which are characterized by the presence of secondary colonizing flora, usually of the shrubby type), old semi-abandoned industrial forest plantations, small plots of land cleared for cultivation (slash and burn horticulture), and vast lots which have been cleared and where different urban settlements and rural facilities of varying size and complexity can be found now. The most important is the "Colonia", a generally small area of agricultural production and livestock keeping. The lands were originally allotted to European immigrant families that arrived in Argentina in the second half of the 19th century. These people and their descendants, still living in these lands, are known as "colonos" [3]

In the course of time this ecosystem has been seriously depleted, and only a very small amount of its original territory remains at present. This depletion has been the consequence of several factors. On the one hand, the development of specific activities – such as the exploitation of timber resources in the rainforest; the substitution of native species for exotic tree species; the building of hydroelectric dams; and agricultural colonization, especially tea (*Camelia sinensis*), *yerba mate *(*Ilex paraguariensis*), tobacco *(Nicotiana tabacum*), and tung tree *(Aleurites fordii*) – has had an impact on this process [4]. On the other hand, what has also contributed to the transformation of the rainforest is the sustained use of its natural resources through hunting and fishing, gathering, and "slash-and burn" agriculture – all of which are subsistence strategies of aboriginal groups in the area [5,6].

In the literature on history, and especially so in the literature on ethnographic studies, the ancestors of the *Mbya *are referred to as the *Kay'gua*, *Kaingua*, or *Caingua *– terms that mean "those who come from the forest" or "those from the *monte*". The terms probably encompass those groups that were not assimilated into the Jesuitical Missions in the XVII and XVIII centuries [7]. According to testimonies collected by León Cadogan [8], the *Mbya *think of themselves as originally coming from the *Yvy Mbyte *(the "Center of the Earth" or the "Center of the World"), a mythical place which they situate in what is now the Departament of *Caaguazú *in Paraguay:

*"(...) the originary country of the Mbya is the Yvy Mbyte, or center of the Earth, situated in what is today the Departament of Caaguazú. It was here that Pa'i Reté Kuaray was born. He was the so-called elder of the two identical twins, and father of all the Guaraní, and he was the child of a god and a beautiful maiden called Ñande Jary (our grandmother). Even today, "he who prays good prayers" can still see the footprints left by Ñande Jary in the sand around Yguá Yvú – the place of spring water. This is in the Yvy Mbyté, (...), where the eternal palm tree Pindojú stands, under which lived the fathers of our people. (...) Foreigners are the yvypo amboaé – those who came from strange lands. God gave them and their mestizo descendants – the jurua or "hairy mouths," that is, the Paraguayans – fields and plains where to raise their cattle, and their horses, and their other animals. God gave the Indians the forests and everything in them, and he made things so the two peoples would live apart. The Paraguayans and the foreigners who settle in the forests are, therefore, usurpers." *[9].

According to Garlet [10], the *Mbya *currently living in the province of Misiones would be the descendants of the *Mbya *from the *Caaguazú *area who started to migrate towards Argentinean territories at the time of the War with Paraguay (1865–1870). This war was the cause (or at the very least the historic moment) of the crossing of the border. Indeed, the very first references to the presence of *Mbya *groups in the province of Misiones date back from 1870 [11,12]. It is from this area that they then moved to the Brazilian states of Santa Catarina and Rio Grande do Sul [10].

According to official sources there are 52 *Mbya *communities in the province of Misiones which are settled alongside national highways 12 and 14, and alongside provincial highway 7. These communities add up to approximately 3700 people.

**Figure 1:**
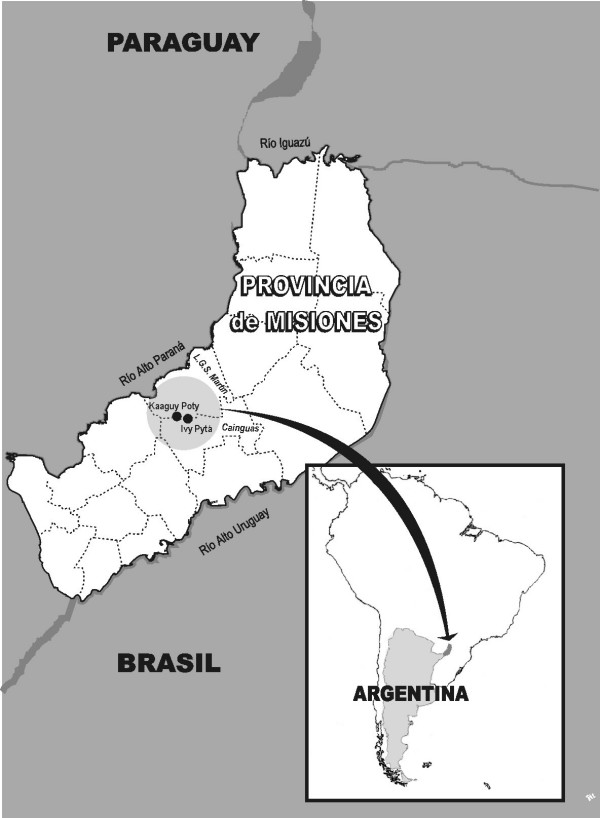
Map.

**Figure 2:**
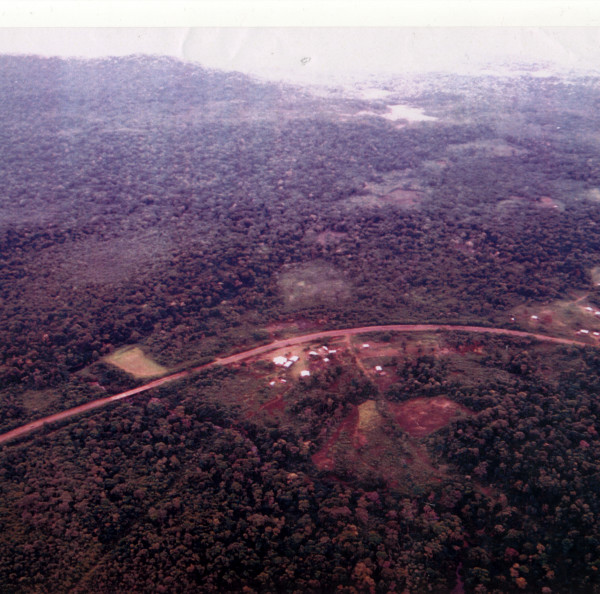
Panoramic view of *Mbya *villages.

The *Mbya *communities under study – *Ka'aguy Poty *(Flower of the Forest) and *Yvy Pytã *(Red Earth) – are settled in the Reserve called *Reserva Privada Universidad Nacional de La Plata Valle del Arroyo Cuña Pirú*. The Reserve is an area located in the basin of the *Cuña Piru *stream which is both, Department of *Libertador General San Martín *and Department *Cainguas *(see Figure 1, 2). In a census carried out by the authors of this study in May 2003, it was determined that a total of 277 people inhabit the area (183 people in the former and 94 in the latter). The members of the *Ka'aguy Poty *and the *Yvy Pytã *communities use the *Mbya *language in their everyday communication and exchanges, but most men, young women, and children attending school also speak Spanish. Some grown-ups speak the Paraguayan variety of *Guaraní *(known as *jopara*), and/or Portuguese as well [13].

## 2. Our approach

Our research has focused on the relationship between human communities and their environment as a result of particular ways of life. The ethnographic approach, which involves thorough research into everyday activities within groups living in the same specific area for long periods, has enabled us to transcend the limitations of macro-analytical perspectives. The relationship between humans and their environment can specifically be identified in those practices which are oriented towards the resolution of everyday problems. We assume that it is in the context of these everyday experiences that the ideas about the environment form and are put to the test.

Daily domestic activities are defined by routines, generated by expectations that are developed over time, and performed in settlements that are in turn outline by these same activities. As such, they offer an adequate starting point from which to consider material, symbolic, and social aspects of human ways of life in different contexts [14].

The purpose of this work is to analize the spatial mobility associated with the searching and gathering of therapeutic resources for treatment of gastrointestinal illnesses. These resources are mainly plants called "poa" in *Mbya *language and "yuyos" in Spanish. Taking into account that *Mbya *strategies for resolution of illnesses episodes are based on the use of this kind of resources, these activities are a good example of how the development of this specific domestic activity brings about patterns of mobility.

The high prevalence of gastrointestinal diseases, like diarrhea or parasite infections and other associated pathologies, for example, anaemia and nutrition deficiencies, leads us to study the local therapeutic strategies to deal with them. The epidemiological significance of these kinds of pathologies and the considerable significance they have in local medical practices justify this decision [7,15]

Two methodological strategies have been used. On the one hand, ethnobotanical interviews oriented to gain knowledge of these resources from both experts and laymen. On the other hand, the course and progress of the treatment illnesses using these resources was followed, either while the illness was being treated at the time, or shortly thereafter. We choose those illness episodies, whose symptoms correspond to "gastrointestinal diseases" from a biomedical perspective.

Male and female adults were interviewed (15 interviewees), and members of our team accompanied them in their gathering trips so as to identify which resources were being used and where they were found, and so as to also collect sample specimens. The informal nature of our interactions with informants during these trips allowed for spontaneous comments and observations having to do with both the spaces of mobility and with those resources which were selected.

As we stopped and observed the sample specimens and collected them for further botanical study, we were able to gather more accurate information on the organoleptic and functional characteristics of the specimens, which in turn allowed us to identify local ways of choosing plant remedies.

In this sense, the trips turned out to be a privileged perspective from which to gather and interpret information about the conceptions the *Mbya *have of their environment and the ways in which they interact with and domesticate it [16].

**Figure 3:**
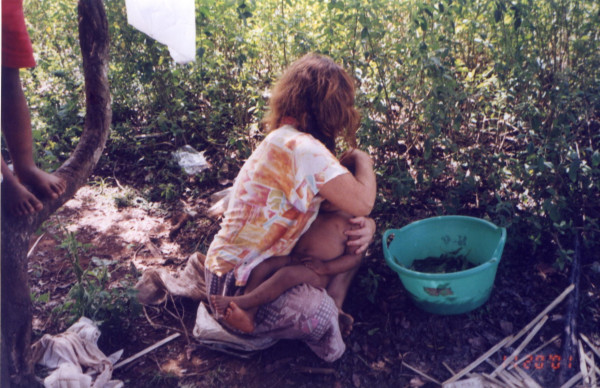
Administration of therapeutic preparation (by head washing).

**Figure 4:**
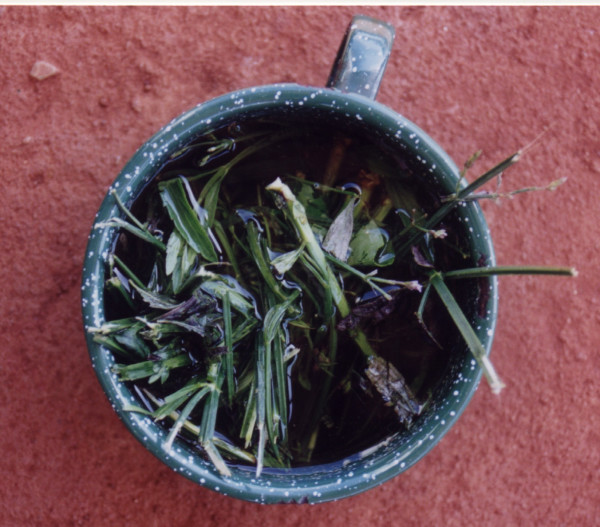
Preparation of medicinal plant.

**Figure 5:**
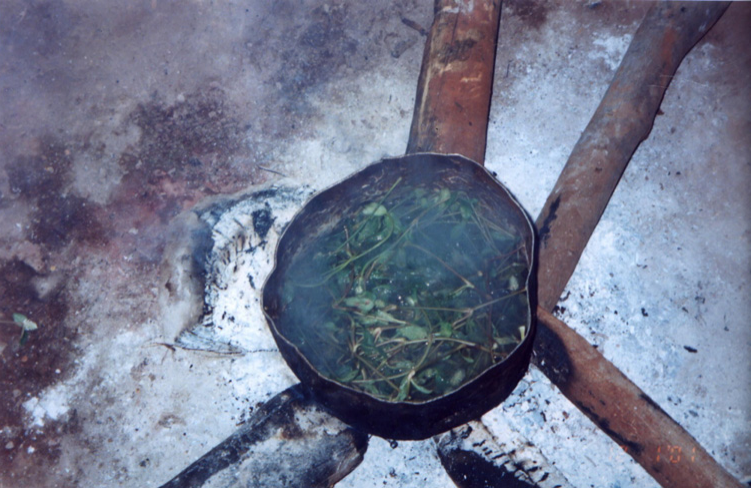
Preparation of medicinal plant.

In the context of the study of 15 episodes of illness, 30 interviews with 23 interviewees were conducted (12 females and 11 males). In 4 cases, we were able to observe how the therapeutic resources were handled, prepared into medicines and then administered (see Figures 3 to 5).

The results obtained by our approach have enabled us to formulate hypotheses concerning these search and gathering trips and concerning the resulting patterns of mobility. They have also enabled us to formulate hypotheses with reference to how the patterns of mobility may affect and influence the design and construction of the *Mbya *space and environment.

## 3. Results and discussion

### 3.1. The activities and their spaces

The way of life of the *Mbya *is the result of the activities they design and perform in the *monte *area – what the *Mbya *call *ka'aguy *(the forest) or *ka'aguy ete *(the true forest or the true *monte*) [17].

*Monte *is the name given in Spanish to those areas of the rainforest where there is a predominance of trees of considerable height and an abundance of vines and epiphytes, as well as a great diversity of animal species. There is a correlation between this biodiversity and a microenvironment diversity which is in turn sustained and transformed by aboriginal communities as they perform their subsistence activities. The *monte *area is thus transformed by human action into different spaces in which there is a peak of human activity.

Close to the villages there are clearings in the forest where the so-called *chacras *(a word in Spanish to denote small farms where horticultural activity is carried out) are found. These small farms or plots for cultivation – called *kokue *in *Mbya *– are distributed around, and usually adjacent to, the dwellings. They do not present a regular pattern, but are rather irregularly shaped, and usually have diffuse and undefined borders. Their dimensions range from between half a hectare and one hectare, and different varieties of corn (*Zea mays*), sweet potato (*Ipomoea batatas*), cassava (*Manihot esculenta*), peanut (*Arachis hypogaea*), squash (*Cucurbita pepo *and *C. moschata*), watermelon (*Citrullus lanatus*), and kidney beans (*Phaseolus vulgaris*), among others, are grown here. At present, fruit trees such as citrus and peach trees have also been introduced. The horticultural activity in these *chacras *is organized around the "slash-and-burn" system, which involves the design and execution of different steps (*macheteada*, or slashing proper using *machetes*; burning; *recoibarada*; sowing and planting *carpida*; and *colecta*, or reaping and harvesting). This specific agricultural technique requires the active involvement of most members of the domestic unit, even the children. In the organization of the horticultural activity, the diverse tasks are assigned in a differentiated way according to sex and age, while members of other domestic units may occasionally take part at different stages of the activity.

The *chacras *are utilized and then left to "recover" when they are no longer as productive, or when the group migrates, or when the group develops alternative economic activities, which results in a new make-up of the area – the "*capueras*", which the *Mbya *call "*kokuere*" (that is, "what used to be "*kokue*" or "*chacra*"). "*Capuera*" is the name given to these cleared areas, noted for the presence of secondary colonizing flora, usually shrubs as well as small trees, which are characteristic of disturbed environments.

The disturbance which gives rise to the *capuera *areas may also be the result of activities performed outside the group. A typical example of such a disturbance may be the building of a highway, in which case the areas adjacent to the road are abandoned. Unless these areas next to the road are used again, vegetation typical of the *capuera *grows there with the resulting development of secondary tropical forest.

The *Mbya *perceive and interpret this process as a transformation of the landscape back into *monte*, a process which goes through subsequent stages. In order to differentiate the initial from the final stages of this successive transformation, some terms are used by the *Mbya*, for instance, *ka'aguy ete *(that is, the primary *monte*), which is for the most part made up of tall trees of a certain height; and *ka'aguy karape *(the *Mbya *for low *monte*,: that is, the area of secondary *monte *make-up), where relatively low tree species and shrub species are to be found.

In this sense, the *monte *areas are not perceived as homogeneous, but consist of different spaces which always refer us back to the intervention of humans in the forest, from the areas such as the *chacras *or the *capueras *described above, to the so-called *trillos *(pathways in the forest resulting from the movement of either animals or humans), from certain plants (cane beds, cane thicket, sources of food for potential prey), to streams or brooks (which are fishing spots and sites of water supply).

It is in the *monte *and in the *capuera *areas where hunting and fishing as well as gathering activities take place [18].

### 3.2. The shaping of the Mbya spaces and pathways in everyday mobility

When the everyday life of the *Mbya *is considered, it becomes clear that patterns of mobility can be identified within a given community as well as between different settlements and villages. Indeed, close observation of those activities which take place within the domestic sphere has enabled us to recognize the commonplace patterns of mobility – each of which is associated with the development of a specific activity – and to identify spaces and pathways shaped as these everyday activities are developed. The members of different households select given spaces in their everyday mobility, and adapt these spaces as they make use of them in daily activities (such as the collecting of water, or activities associated with personal hygiene), or in activities such as the manipulation of species from the *monte *area, which are then carried and transplanted close to the dwellings for dietary or therapeutic purposes.

The dwellings are surrounded by an area free of all vegetation called *patio *in Spanish or *oka *in *Mbya*, which is where women and children typically tend to spend most of the day, and where members of different households gather and meet. It is here that the so-called *fogones *are placed – fires which are kept constantly burning and which are used by the members of the household to heat water, to cook, or in the manufacturing of handicrafts. Shrubby vegetation characteristic of the *capuera *in turn surrounds these *patio *areas. Fruit trees and other plants like the local species known as *güembe*, *pindo*, or *araza *(which are brought specially from the "monte" area to be planted here) are occasionally interspersed with this shrubby vegetation.

A literal web of paths called *tape *connects the *patio *area with the other parts of the village: the school, the community meeting hall, the stream (or *ỹy ak*ã in *Mbya*), the *monte *(or *ka'aguy*), and the *chacras *(or *kokue*). The paths also serve the purpose of connecting the different houses with one another; notably, the houses of extended family members are usually placed close to each other, and can even be arranged around the same central "*patio*" area when the family is especially large. Additionally, there is a pathway which runs parallel to the road, connecting both communities (see Figure 2).

While these paths mentioned above are human-made, some of the paths found are the result of the movement in the forest of animal species such as wild boar (*jabali *or *kochí), tatú*, or deer (*guachu pytã*). These paths are called *trillos *and they usually follow water courses or vegetation areas where animals of prey prefer to feed. Recognition and identification of these paths made by the movement of animal species in the forest is central in hunting trips and determines the choice of specific locations to set traps and snares.

**Figure 6:**
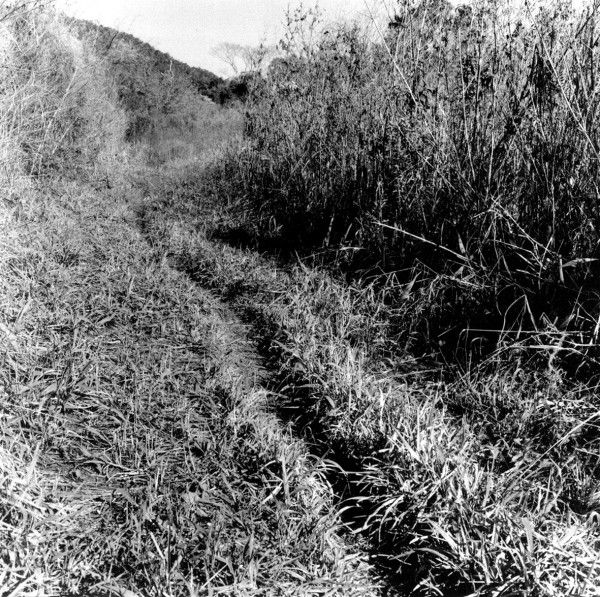
Pathway through the *capuera*.

**Figure 7:**
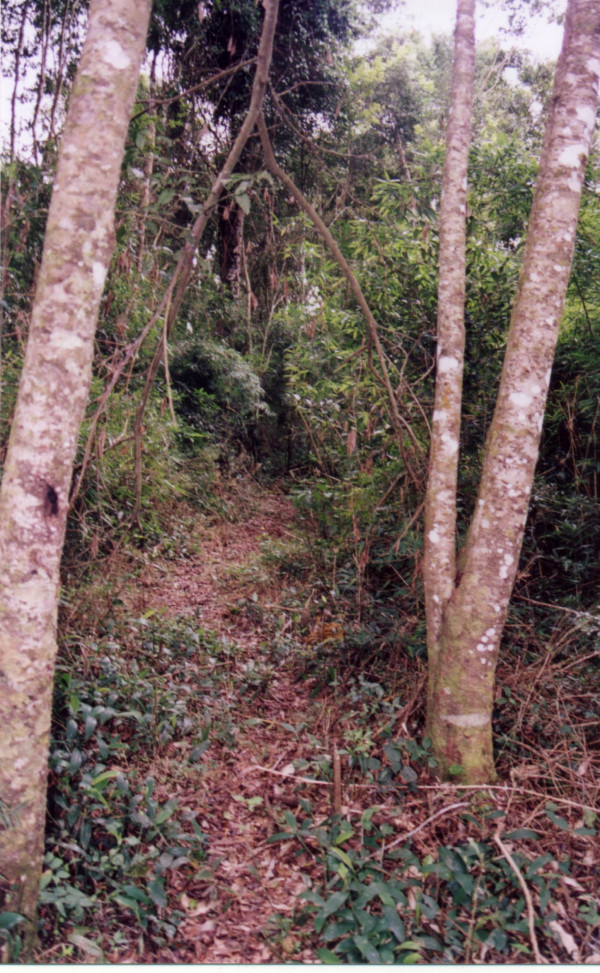
Pathway in the *monte*.

Those specific paths to and from water sources are good examples of alternate uses which range from communal to individual, from public to private. One case in point is the stream. The stream is a meeting place for women and children, more often dwelling in the same household or in households which are located nearby, who tend to use either branches from the main stream or else specific spots along its course to bathe in or to perform activities of personal hygiene in relative privacy. The stream is also used individually by hunters during day-long hunting trips, who take advantage of its branches to construct drinking spots alongside paths where their snares are set. These snares then function as individual markers which "personalize" the path, as it were. But, while the path is in this way personalized and marked by specific individuals, it nevertheless remains available for other people to use. On the whole, a path cleared for a specific purpose may eventually be put to other uses. Thus, a web of pathways going in different directions, and opened by different agents at different points in time and with different purposes in mind, shapes the dynamics of the daily life of these communities (see Figures 6, 7).

Through this web of pathways it is possible to perceive and understand the transformations in the environment which are usually very subtle and overlap this movement. With regard to the trips and movements the *Mbya *make in their search for plant resources to treat gastrointestinal illnesses, our research attempts to make it evident that this approach is truly effective.

### 3.3. The search for therapeutic resources

The methodological strategies used in our study (see section 2.1) enabled us to gain knowledge on the use of 29 different medicinal plants. References to each of these species, the names by which they are known in both *Mbya *and Spanish; the part of the plant used; location and how it is collected and prepared are recorded in Table 1.

**Table 1:** Plant resources employed in the therapy of parasitosis and related diseases

**Disease**	**Plant designation**		**Scientific name and family**	**Preparation**	**Part used**	**Origin and obtaining**
	***Name in Spanish***	***Name in Guaraní or Mbya***				
Diarrea **(*Gerachy*)**		Ka'a re*	*Chenopodium ambrosioides *L. (Chenopodiaceae)	Infusion	Aerial part	New World (NW): wild, surroundings of the home ***Capuera Monte***
	Marcela	Poty ju*	*Achyrochline satureioides *(Lam.) DC (Asteraceae)	Infusion	Aerial part	NW: wild, surroundings of the home ***Capuera***
		***Jate'i ka'a***	*Achyrochline *sp. (Asteraceae)	Infusion	Aerial part	NW: wild, ***capuera***
		Guavira*	*Campomanesia xanthocarpa *(Mart.) Berg. (Myrtaceae)	Infusion	Leaves	NW: wild, ***monte***
		Parí-paroba*	*Piper mikanianum *(Kunth,.) Steud (Piperaceae)	Infusion	Leaves	
	Guayaba	***Araza***	*Psidium *sp. (Myrtaceae)			NW: wild, from ***monte ***but cultivated in the surroundings of the home
	Achicoria		*Cichorium intybus *L. (Asteraceae)	Concoction	Root	Old World (OW): adventitious in Argentina ***capuera***
		Jabrandi*	*Pilocarpus pennatifolius *Lem. (Rutaceae)	Infusion	The whole plant	NW: wild, ***monte***
Stomach ache **(*Hy'erasy*)**		Guavira*	*Campomanesia xanthocarpa *(Mart.) Berg. (Myrtaceae)	Infusion	Leaves	NW: wild, ***monte***
	Pitanga	***Añanga piry***	*Eugenia uniflora *L. (Myrtaceae)	Infusion	Leaves	NW: wild, ***monte***
	Marcela	***Poty ju ***	*Achyrochline satureioides *(Lam.) DC (Asteraceae)	Infusion	Aerial part	NW: wild, surroundings of the home ***Capuera***
	Doradilla	***Amambai***	*Aneimia *sp. (Schizaeaceae)	Infusion	The whole plant	NW: wild, ***del monte***
Parasites **(*Tacho*)**		Ka'a re*	*Chenopodium ambrosioides *L. (Chenopodiaceae)	Infusion	Aerial part	NW: wild, surroundings of the home ***Capuera Monte***
	Raíz de perejil		*Petroselinum crispum *(Apiaceae)	Not registered	root	OW: cultivated ***chacra***
	Horqueta	***Sapyragy***	*Tabernaemontana catharinensis *(Apocynaceae)	Concoction	Stem bark	NW: wild ***monte***
	Cangorosa	Yvyra rapo ju*	*Maytenus ilicifolia *Reiss. (Celastraceae)			
		***Guaporovity***	*Plinia rivularis *(Myrtaceae)	With cold water	Stem bark	NW: silveste, ***Del monte***
		***Yvyra-ro***	*Pterogyne nitens *Tulasne (Fabaceae)		Stem bark	NW: wild, ***l monte***
	Verbena	***Guachu ka'a***	*Verbena intermedia *Gill. Et Hook. (Verbenaceae)	Infusion	Aerial part	NW: wild, surroundings of the home ***Capuera***
	Pitanga	***Añanga piry***	*Eugenia uniflora *L. (Myrtaceae)	Infusion	Leaves	NW: wild, ***monte***
		Jabrandi*	*Pilocarpus pennatifolius *Lem. (Rutaceae)	Infusion	The whole plant	NW: wild, ***l monte***
		***Typycha hu ***	*Sida rhombifolia *L. (Malvaceae)	Infusion	Root	NW: wild, surroundings of the home ***Capuera***
		***Pipi guasu Sapyragy***	*Petiveria alliaceae *L. (Phytolaccaceae)	Concoction	Root	NW: wild,***monte***
		Ka'api kachi*	*Kyllinga *sp. (Cyperaceae)	Concoction	Aerial part	
		***Tembetari***	*Zanthoxylum hyemale *(Rutaceae)		Leaves	NW: wild, ***monte***
	Rabos	***Yvyra kachi***	*Lonchocarpus *sp. (Fabaceae)	Concoction	Stem bark	NW: wild,***monte***
	Siete capotes	***Ñandú apycha***	Not collected	Concoction	Leaves	NW: wild,***monte***
Rotating stomach **(*Kambi ryru jere*)**	cáscara de mandarina		*Citrus reticulata *(Rutaceae)	Burnt and then prepared in infusion	Fruit rind	VM: cultivated ***Chacra. colonia***
	manzanilla		*Matricaria recutita *(Asteraceae)	Concoction (in combination with other plants, as seen below)	"Center of the flower" (only the tubulous flowers of the inflorescence)	OW: cultivated in the surroundings of the home ***Capuera***
Strong abdominal pain.	Eucaliptus		*Eucalyptus *sp. (Myrtaceae)	Infusion (In the water for ***mate***).	Leaves	Australia: cultivated ***Colonia ***
Stomach sickness		***Jaguane ka'a***	*Coronopus didymus *(Brassicaceae)	Concoction	Leaves	NW: wild ***capuera***
Diarrhea caused by ***tacho***		***Jaguarete ka'a***	*Baccharis *sp. (Asteraceae)	Concoction (in combination with other plants, as seen below)	Not registered	NW: wild ***capuera***
Diarrhea with blood		***Ñandyta***	Not collected	Concoction	Stem bark	Wild, ***Monte***
Diagnoses	tabaco	***Pety***	*Nicotiana tabacum *(Solanaceae)	Burnt and smoke used for diagnoses and first steps of the therapy	Leaves	NW: cultivated ***chacra***

* resources mentioned both in ethnobotanical interview as well as in the following of illness cases

Several conclusions can be derived from this information. Among all the studied species, the only one all subjects agreed is effective against intestinal parasites is what the *Mbya *call *ka'a re *– *Chenopodium anthielminticum*, which is known in Spanish as *paico*, which is used worldwide as an anthelmintic and an antispasmodic [19,20]. The *Mbya *collect the *ka'a re *in the *capuera *areas adjacent to the houses as well as in the *monte*. Other species are alternatively used in the treatment of parasites according to both availability and personal preferences. For instance, two of the experts, called in *Mbya "poro poãno va'e"*, interviewed in the course of our study make a reference to the *tembetarí*, a plant not mentioned by any other members of the community, as effective in the treatment of parasites.

The plants mentioned in the ethnobotanical interviews are obtained by means of collecting them either in the "*monte*" area (14) or in the "capuera" next to the houses (5). As a rule, those resources to be found in the *monte *are collected by the experts (*poro poãno va'e*), while those found in the areas adjacent to the houses are collected by the members of the domestic unit (DU). Once the plants are obtained, adult members of the DU are in charge of both preparing and administering the medicine. Whereas some of the plants may be combined to make a specific medicine, most plants are used separately and independently of others.

Among the different resources used to treat parasitic illnesses, there are recurrent references to how certain species are effective in the treatment of two of the most frequent parasites, those called *tacho pytã *and *tacho ovy *in *Mbya*, which can be detected and discerned macroscopically, and which in principle might seem to correspond to *Ascaris lumbricoides *sexual dimorphism [4,21]

As a rule, a medicinal plant's effectiveness is associated with its organoleptic characteristics, especially its olfactory and gustative characteristics. A strong smell and bitter taste in a plant, for example, is associated with the attribute of that species to destroy parasites. The search for these plants may require separate trips in different directions, as they may be dispersed and distributed loosely. It is by studying and following the course and progress of the treatment of illness that we have been able to gain knowledge of these voyages in search of therapeutic resources.

In the study we found that most of the relationships existing among those subjects involved in 15 episodes of gastrointestinal illnesses concerned the circulation and use of plant resources. These interactions occur during the search, during the manipulation or processing, and during the administration of the following: tangerine peel, *sapyragy*, eucalyptus tree, *guaporovity*, tobacco, *jaguane ka'a*, *jaguarete ka'a*, *jabrandi*, *ka'are*, *kapi'i kachi*, *pari paroba*, *guavira*, chamomile (named locally in Spanish, *manzanilla*), *marcela*, *ñandyta*, parsley root, and *yvyra rapoju*. The areas where these are collected include the *colonias*, the *monte *area (5 of them), and the *capuera *areas next to the houses of those people who prepare and administer the resources (18 of them) (see Table 2).

**Table 2:** Resources mentioned in illness cases studied

**Episode/case**	**Involved People**				**Processing**		**Space of obtaining/resources**		
***Number***.	***Ill person ***	***Relative***	***Expert***	***Others***	***In the UD***.	***Out of the UD***.	***colonia***	***monte***	***capuera***

1		X			X				***ka'are ***
		X			X				***Marcela***
		X	X		X				***Marcela***
		X	X		X				***Raiz de perejil ***(near an UD from the other community)
2		X	X		X				***Marcela, jaguarete ka'a, ka'api kachy manzanilla***
3			X	X		X			***Manzanilla, cáscara de mandarina***
4		X			X				***Marcela***
5		X	X			X			***ka'are, manzanilla ***
		X			X				***ka'are***
		X			X				***yvyra rapo ju (cangorosa*)**
6		X			X			***Jabrandi***	
7		X	X		X		***Eucalipto***		
		X	X		X				***Jaguane ka'a***
8		X			X			***Ñandyta***	
9		X	X		X		***Mandarina***		
10		X			X			***Guaporovity***	
11			X			X			***Tabaco***
		X	X		X				***busca ka'are***
12	X				X			***Sapiragy***	
13			X			X			***Tabaco***
			X			X			***ka'are***
14			X		X				***Tabaco***
			X		X				***¤***
15		X			X				***Marcela***
		X			X			***Guaporovity***	

¤ For this case the expert gives ***guavira, pari paroba, manzanilla ***and ***ka'api cachi***. As those resources are not easy to obtain, people make the decision of attending hospital.

If we focus our attention on the spaces where these resources are manipulated after being collected, we observe that most of them (18 of all 25 resources studied) are processed and administered in the sphere of the household. Closely related to this, we have also noticed familial relations are typically involved in this process, even though some cases have been reported where these relatives were acknowledged by the household or community members as experts. Only 5 of the total number of resources studied involved spheres of processing other than the household proper, and these were related to consultation with local experts.

Multiple pathways were identified in the movements to and from the *capuera *and the *monte *areas, and, interestingly enough, no two subjects reported using the same pathway to get to the same place.

Those resources which are most frequently used are found in the *capuera *area adjacent to each house. Close to these areas, there are paths that stretch to the *chacras*, where resources such as parsley are collected, and further into the *monte *area, where the larger species are to be found (see Figures 6, 7). The *Mbya *also go into the lands of the *colonos *to get those resources that are solely grown there (such as eucalyptus tree or tangerine tree).

The information in Table 1 – species and the spaces where they are found and collected – shows differences between data obtained in the context of the case studies and data obtained from the ethnobotanical interviews. Information obtained by means of ethnobotanical interviews, shows that more than half of the 19 plants mentioned are said to be found and collected in the *monte*, while the rest are said to be found and collected in the *capuera *areas. The relationship, however, is reversed when the results from the case studies are considered. It turns out that over half of the species used out of a total of 17 are obtained in areas other than the *monte *(*capueras*, *chacras *and *colonias*), and we have observed that most resources come from the *capuera *areas, with only some cases in which the resources originating from the *monte *are then grown either in the areas surrounding the houses (the case of *ka'a re *and *araza *for example) or in the *chacras *(for instance parsley). The fact that there are references to the uses and properties of therapeutic resources which are exotic to the forest environment is evidence that the *Mbya *resort to new alternatives in the treatment of certain illnesses. This can be demonstrated best when the possibility to obtain traditional resources presents special difficulties. There is a sharp contrast between the strenuous and time-consuming nature of the process to obtain therapeutic plant resources from the "*monte*" and, on the other hand, the relative ease with which resources may be accessed by the *Mbya *in other spaces.

As can be seen in case number 14 in Table 2, the expert gives advise for the preparation of an infusion made from *guavira*, *pari paroba*, chamomile or *manzanilla*, and *ka'api kachi*. Given the fact that 3 of these resources are found in the *monte *area far from the village, and that obtaining them will involve added difficulties (the distance from the village; the effort involved; the expertise and skills needed to locate these resources), the decision is made to take the person who is ill to the local hospital.

As is in different cases in other contexts involving the handling of natural resources, when the strategies to treat gastrointestinal illnesses are justified, we find that an important stress is placed upon the generational factor. It is indeed the elders in these communities who possess a wider, more complete knowledge of the diagnosis and treatment of disease in general and, therefore, of the variability of useful resources for each disease, which becomes evident in the results obtained from the ethnobotanical interviews. However, different members of the DU – especially in the case of the younger members – choose those resources which are the easiest for them to gather, as is clear from the narratives obtained from the case studies. The possible reasons accounting for these changes in how therapeutic resources are handled include a discontinuous transmission of the knowledge and the practices related to the use of these resources, as well as the depletion of the *monte *areas. The former makes it difficult for younger people to identify and collect species in the *monte*, while at the same time it can be impossible for the elderly to access *monte *areas due to the physical strain and effort involved in doing so. The depletion of the *monte *implies that certain species will not be so widely or easily available, with the consequence that the strategy of planting or growing some of these species next to the houses becomes more frequently adopted, and which in turn makes it easier for the members of the DU to have access to these resources.

While those members of the community who are acknowledged as experts refer to a number and variety of possible therapeutic resources supposedly available for the treatment of gastrointestinal diseases, the study of actual cases of illness shows that only a low percentage of these resources are put to real and effective use (36%).

Additionally, a contrast exists between the references of experts to the "*monte*" or "*ka'aguy ete*," (and to a lesser extent the "*capuera*" or "*kokuere*"), as the only relevant spaces to obtain plant resources, and the actual diversity of environments (the "*monte*" and the "*capuera*," but also the "*chacras*" and the "*colonias*") where these resources are in fact sought for and found in order to treat diseases. Based on their knowledge and interests, individual members of the community choose to take a given path or clear a new one, and/or decide to plant otherwise wild resources around their houses. The daily decisions that members of the community take are guided by each individual's assessment of how valuable a resource may be according to the place where it grows. Likewise, these decisions are guided by each individual's recognition of spaces as valuable in order to find the necessary resource within it.

## 4. Final considerations

### Mobility and landscape

Processes of spatial mobility among the *Mbya *are of interest in anthropological and ethnobiological studies, as these processes are related to transformations in the landscape and the environment. Despite this, ethnographic literature usually focuses on the mobility of *Guaraní *communities from the perspective of population dynamics on a regional scale [13,22-24].

These views acknowledge the sociological aspects of a complex phenomenon which evidences how in these processes of spatial mobility the relationship that the *Mbya *have with the *Monte *is a core subject, both historically and at present.

The lifestyle and, especially, the economy of the *Mbya-Guarani*, have been modified over time as a result of the interactions with other American ethnic groups and with Europeans during the long process of conquest and colonization. The colonization of the forest in the province of Misiones is based on different kinds of economic activities (i.e. industrial-type cultivation and the exploitation of timber trees) and has gradually reduced and modified the features of this habitat affecting the territory and culture of the native communities.

Consequently, the *Mbya *characterised by their large scale migrations, that nowadays would be considered transnational, exhibit at present a sedentary tendency, while simultaneously searching through the socio-environment for conditions that make the development of their traditional activities possible.

Indeed, at present as well as in the past, the *monte *is the background setting where the *Mbya *displacements become significant. This is what is called "Guaraní horizon" in archaeological literature [25] – a setting that is characterised by bio-geographical and cultural features which are used to form a map outlining the location and positioning of these ethnic groups. Yet, very little research is devoted to actually exploring the characteristics and dynamics of this mobility as a constitutive aspect of the life and subsistence strategies of the *Mbya *people.

Our ethnographic research on the subsistence activities which are performed in the domestic sphere of two *Mbya *villages has enabled us to recognize patterns of mobility on a micro-scale. The daily trips of both men and women in these communities are the result of a multiplicity of pathways articulating and unifying spaces such as the *monte*, the *capuera*, or the *chacra*. These spaces, moreover, are designed by these subsistence activities.

In the case of the gathering of plant species as therapeutic resources, we have observed how the spaces and the pathways are outlined by the individual. It is the individual who daily recognises those spaces – or micro-environments – where the collecting of medicinal plants is or can be performed. It is also the individual who daily perceives and values these micro-environments – either in terms of the possibility to find the necessary resources, or in terms of how effective these resources might be depending on where they are found or grown. Guided by their own personal knowledge and interests, each individual chooses either to take certain established pathways, to clear a path where there was none or to ensure the availability of specific natural resources by planting and growing them next to their house. In this sense, we found that the most frequently used therapeutic resources were those from spaces adjacent to the dwellings (namely, in the *capueras *and *chacras*), which accounts for the increasing importance attached to these spaces as a source of medicinal plants – both for their consumption within the household, and for their commercialization for profit [26]. As use of these plants increases (with the *Mbya *and others as the agents of this increasing use), so does the process of planting and growing these plants in the *capueras *and *chacras*, which constantly configures and reconfigures the environment of the *Mbya *and their setting. This literally brings the *monte *closer to the *Mbya capueras *and *chacras*.

Both differences in personal itineraries and age also account for the transformations of the *Mbya *setting. Indeed, several factors contribute to the fact that the *Mbya*'s knowledge and use of "*monte*" species is smaller than it used to be. These factors include processes of socialization and an increasing contact of the younger generations with environments that are modified by the intervention of groups other than the *Mbya *themselves – in particular their access to formal education and health services.

Even though, in the course of our research, we have observed that those spaces involved in the obtaining of therapeutic resources were diversified, it is interesting to point out that, in the way the *Mbya *view the world, both the *chacras *and the *capueras *are perceived as transformations of the *monte *– spaces where the *monte *has been transformed or modified in some way, but where it is still "*monte*" area. It is in this sense that the vernacular categories that refer to these modified spaces – *ka'aguy ete*, *ka'aguy karape*, *kokue*, *kokue re *– are all terms that reassert the sequential and evolutionary nature of the *monte *– the *ka'aguy *or *ka'aguy ete*, – in that all of them refer to stages or phases in the transformation of a single space as a consequence of human intervention.

The notion of *monte *as a pristine space, unaltered by humans, is used more often than not in reference to spaces which are increasingly removed from human dwellings. The pathways, that were originally designed to reach the faraway *monte*, today keep the *monte *closer and closer to the dwellings. We have in this sense observed what can be called an opportunistic and multiple use of pathways. For example, hunting prey or checking a snare may present itself as a good chance to also collect plants. Or conversely, the need to collect resources for the treatment of a given illness may be used to also collect other resources, as well as to set traps and snares. As a consequence of this, the diverse paths not only help to link different spaces – what we have previously called "micro-environments" – but they also constitute a space in themselves. Pathways are thus a space which serves the purpose of articulating other spaces, a space whose characteristics become relevant as they relate to achieving the specific goals of specific activities.

In the everyday life of the *Mbya*, the notion of forest is realized as a space that is the product of human activity, not as a pristine reserve of wild species. This notion is in agreement with what Balèe [27] pointed out when he characterized the relationship between Amazonian societies and the forest environment. Balèe introduces here the concept of "anthropogenic forest" – a notion also referred to by Rival [28] – which is the result of centuries of aboriginal interaction within its rainforest environment, and which reflects human activity insofar as human activity modifies both the distribution of different species and the associations those species have with one another. This modification results in a configuration of the environment which is closely connected with those values the community considers central to their way of life [29,30].

Both the narratives offered by different members of the community during our interviews on the one hand, and our direct observations as they manipulate different plant resources during their search trips on the other, have provided us with significant information about the strategies used by the *Mbya *to domesticate the *monte *[27].

## Authors' contributions

MC and MRM have made substantial contributions to conception, design, analysis and interpretation of data; they also have been involved in drafting the manuscript, revising it critically and have given final approval of the version to be published. MLP has collected and identified medicinal plants, and has participated in ethnobotanical and ethnoecological data collection and analysis. CR has made substantial contributions to acquisition of observational data, analysis and interpretation of data; and has been involved in drafting the manuscript. AS has made substantial contributions to acquisition of data, analysis and interpretation of data; and has been involved in drafting the manuscript. LT has made substantial contributions to analysis and interpretation of data and has been involved in drafting the manuscript. All authors read and approved the final manuscript.
